# Focused neurological assessment to differentiate essential tremor from functional tremor

**DOI:** 10.1055/s-0044-1788267

**Published:** 2024-07-29

**Authors:** Thiago Trajano da Silva, Igor Vilela Brum, Isadora Santos Ferreira, Jacy Bezerra Parmera

**Affiliations:** 1 Universidade de São Paulo, Faculdade de Medicina, Departamento de Neurologia, Grupo de Distúrbios de Movimento, São Paulo SP, Brazil.

**Figure 1:**
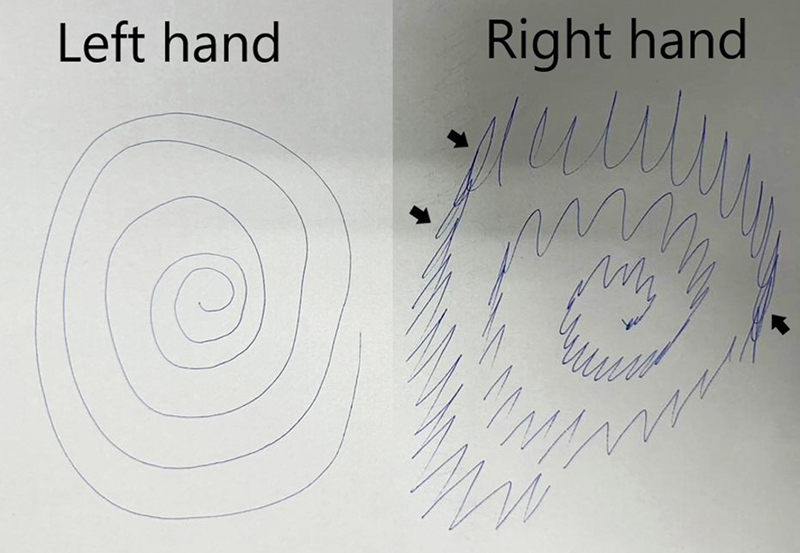
Archimedes' spiral drawing of a patient with functional tremor showing frequency changes and the distinctive “stretched slinky sign.” Arrows indicate the areas of looping.

A 64-year-old female patient presented with a 20-year history of right upper limb tremors, significantly affecting her daily tasks. She was previously diagnosed with essential tremor and reported no response to propranolol (maximum tolerated dose of 80 mg/day). The neurological examination revealed amplitude and frequency variability, distractibility ( Video 1 ), entrainment, and distinctive looping patterns in the Archimedes spiral drawing and handwriting ( Figure 1 )—a phenomenon known as the “stretched slinky sign,” described in a previous study as distinctive of functional tremor, although it has not been formally assessed in control groups. 1 The diagnosis of functional tremor was made based on these positive signs, emphasizing the importance of a focused neurological examination to distinguish it from other tremor disorders. 2 3

**Video 1** Focused neurological assessment of functional tremor. The Archimedes spiral drawing with the right hand reveals variability in tracing frequency and the distinct looping pattern, a phenomenon known as the “stretched slinky sign.” The left hand is unaffected. When tasked with writing her name, the patient also demonstrates looping patterns. When instructed to keep the pen close to the paper while counting from 20 to 1, tremor frequency and amplitude variations are evident, indicating distraction during cognitive tasks. There is an absence of resting, postural, and intentional tremors. (Available at: https://www.arquivosdeneuropsiquiatria.org/wp-content/uploads/2024/06/ANP-2024.0097-Video.mp4 )

